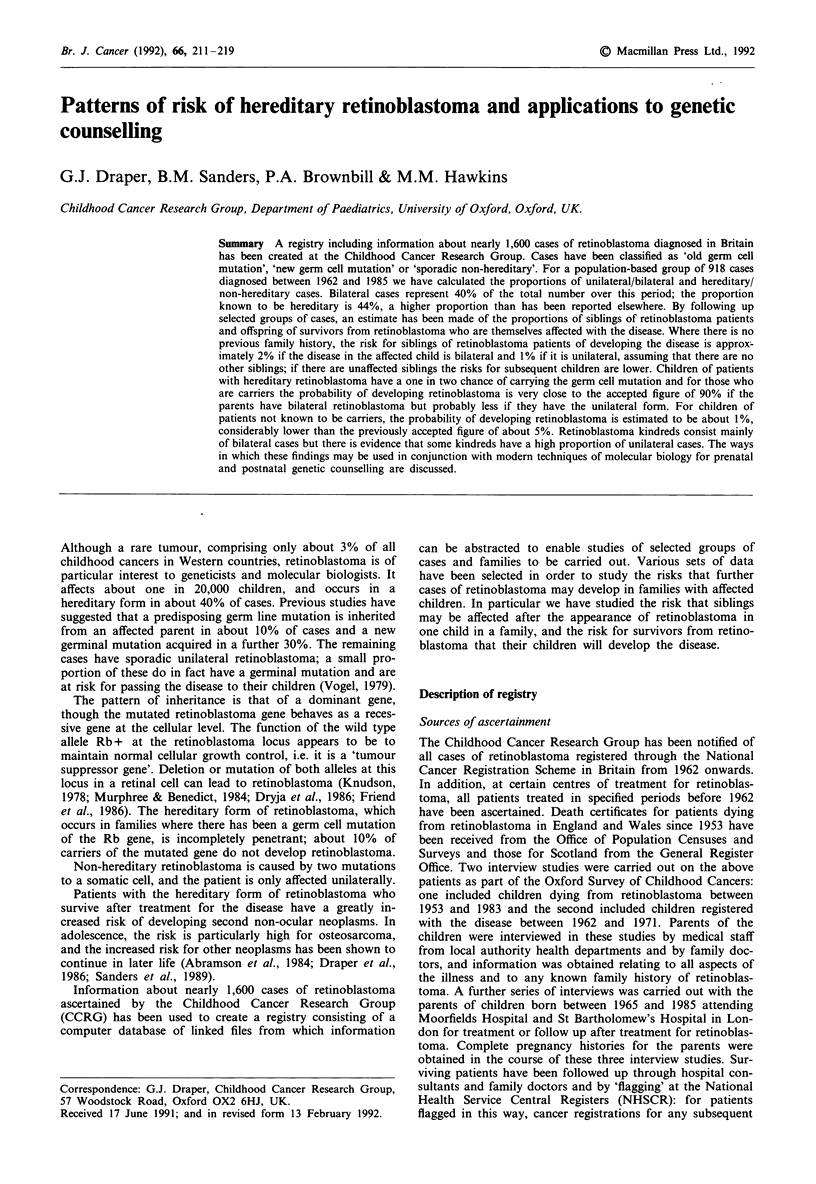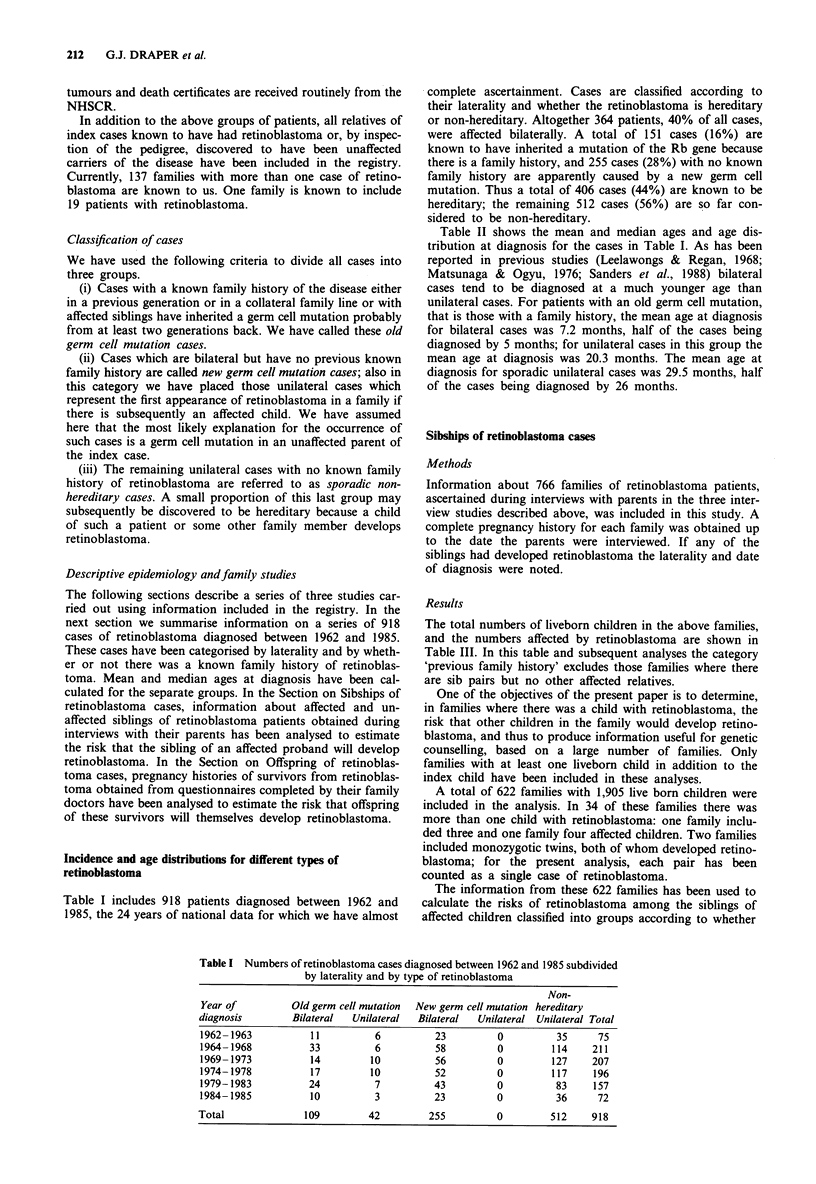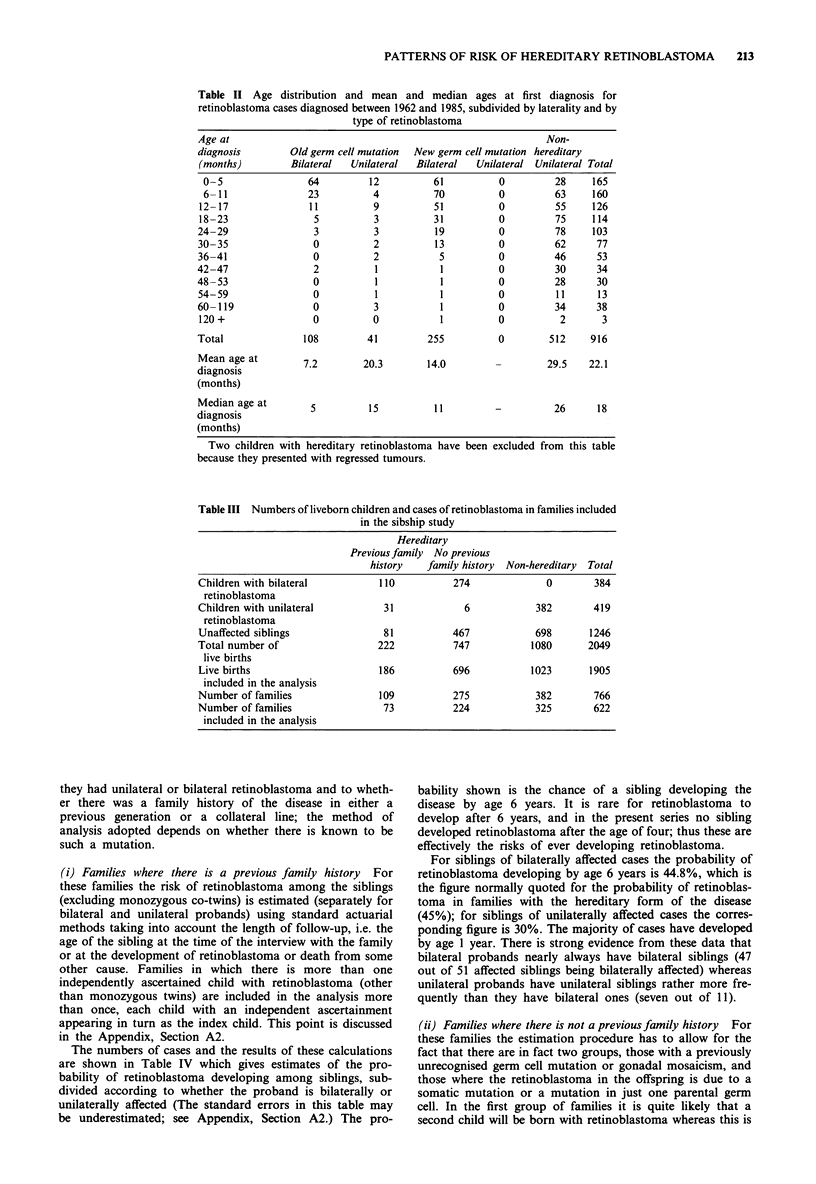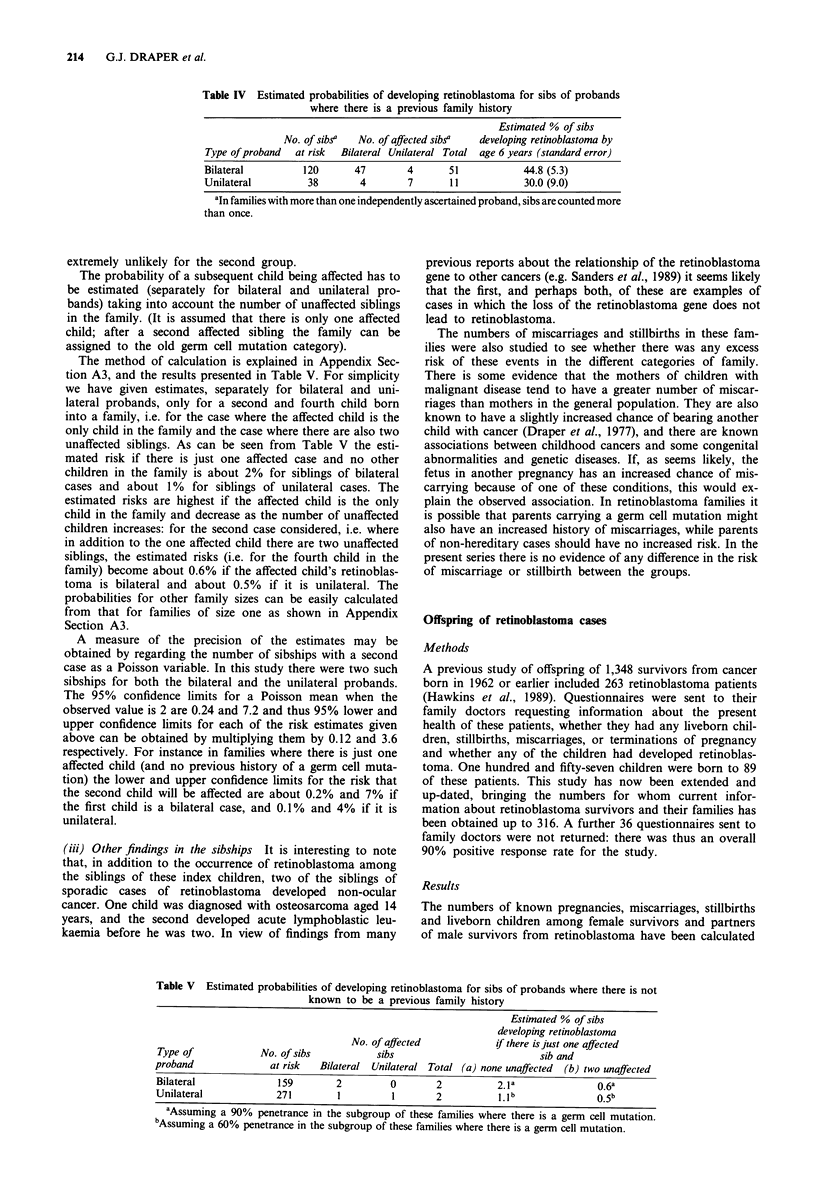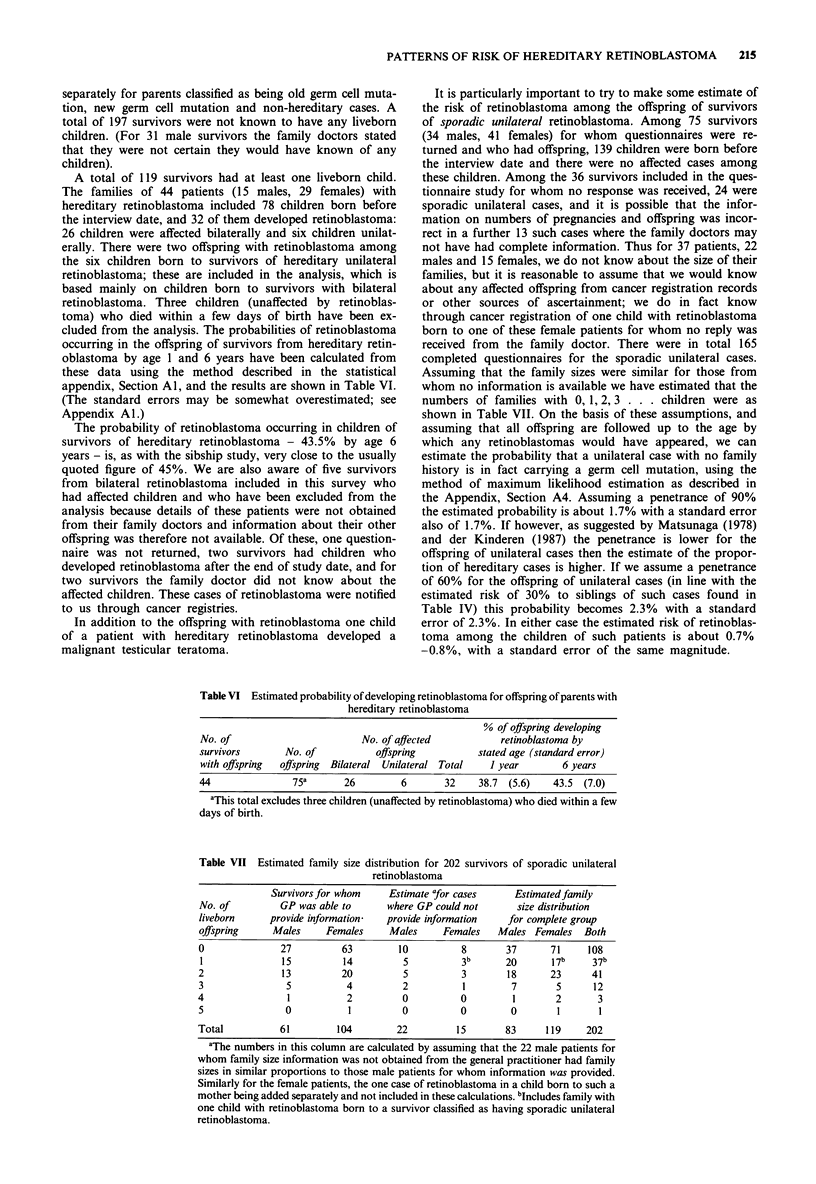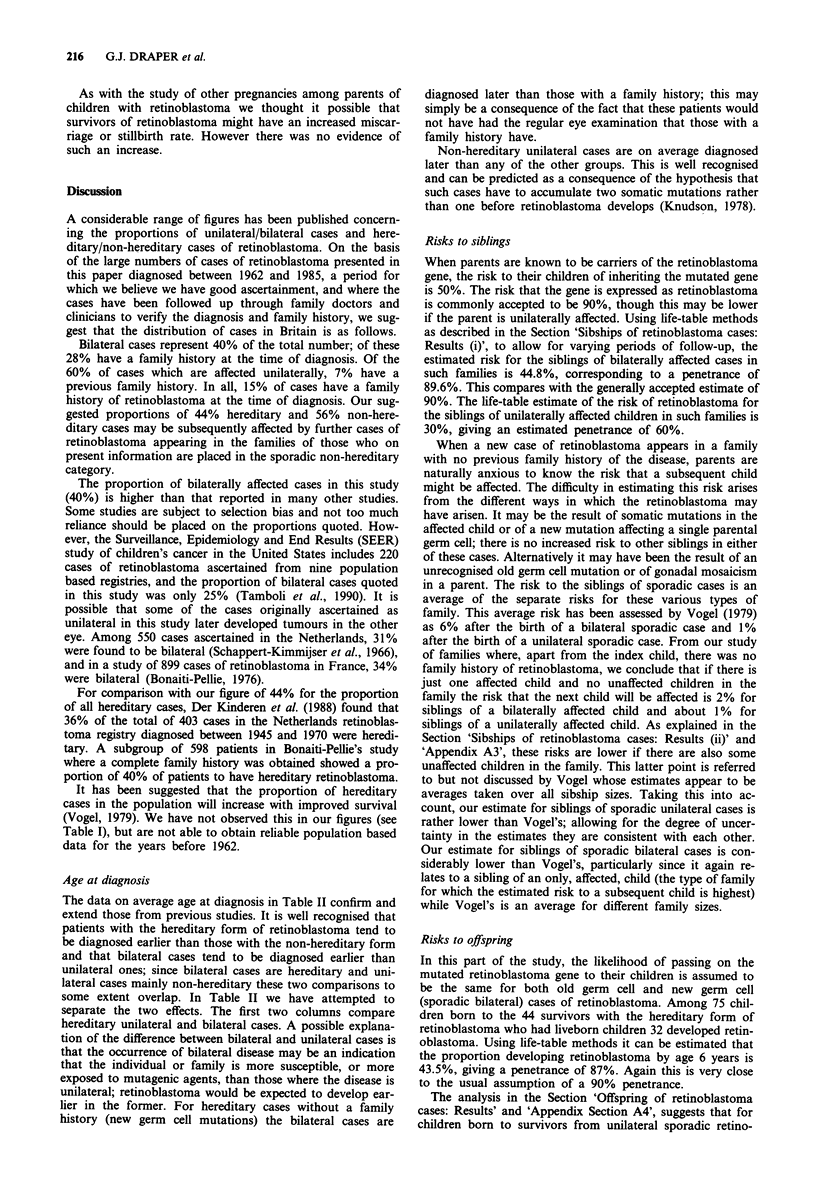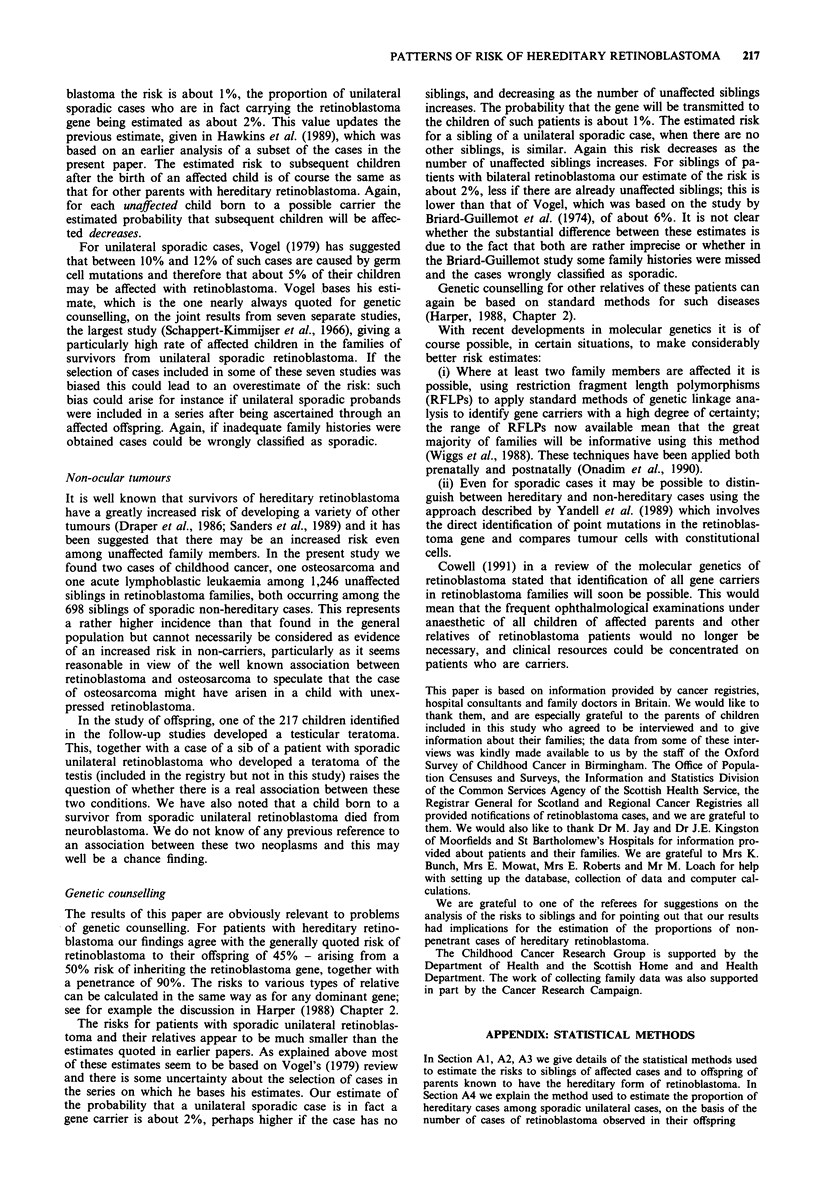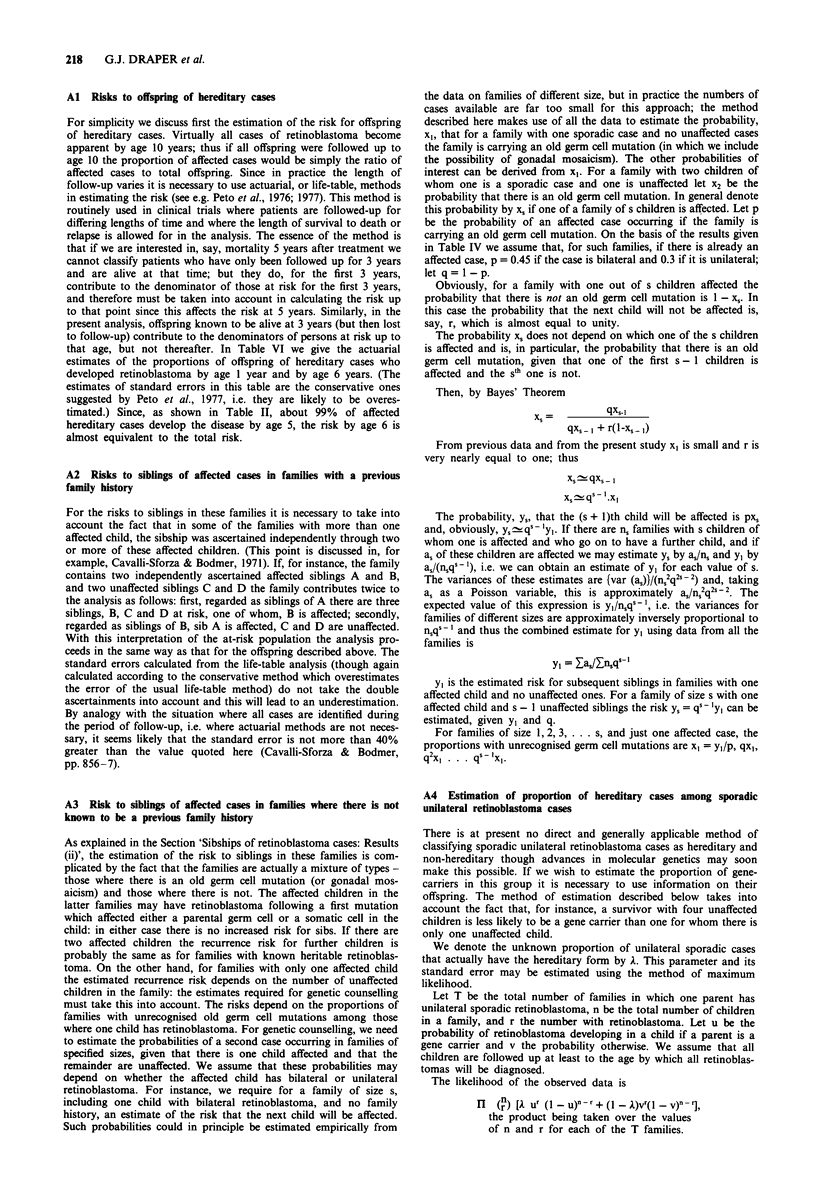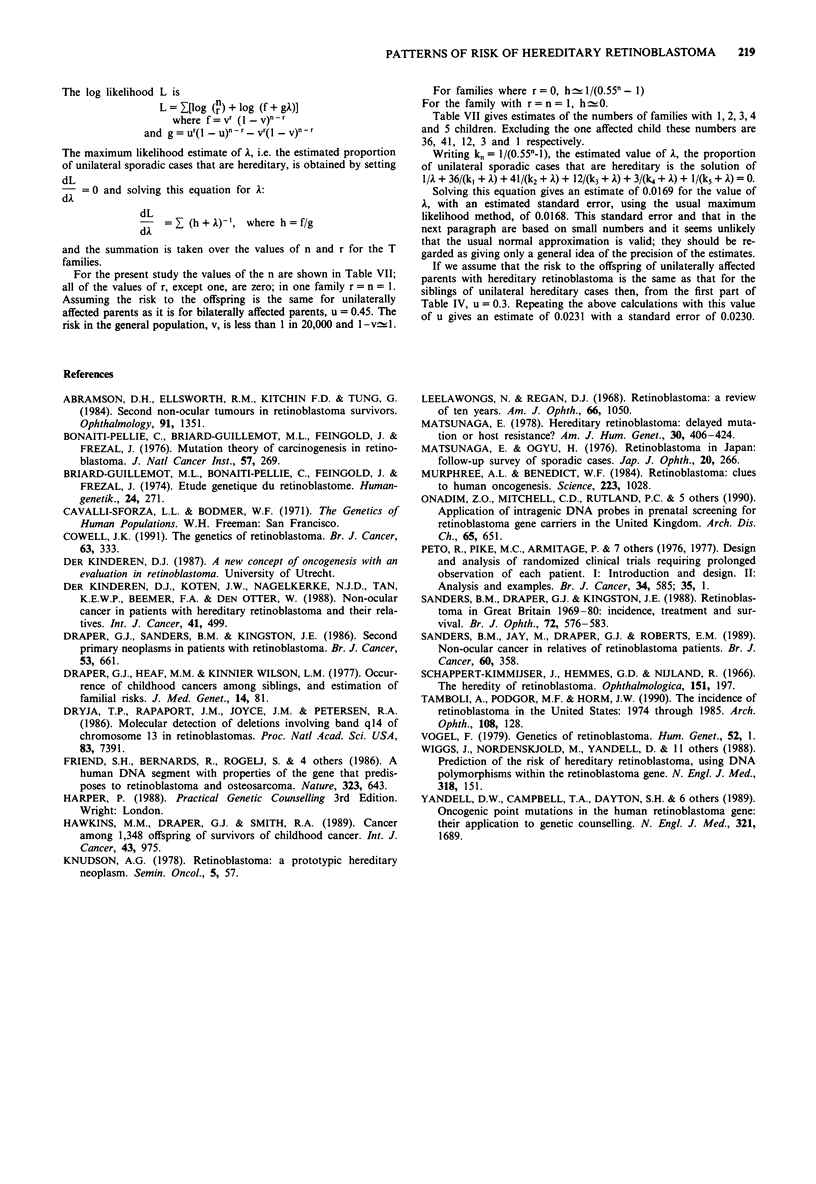# Patterns of risk of hereditary retinoblastoma and applications to genetic counselling.

**DOI:** 10.1038/bjc.1992.244

**Published:** 1992-07

**Authors:** G. J. Draper, B. M. Sanders, P. A. Brownbill, M. M. Hawkins

**Affiliations:** Department of Paediatrics, University of Oxford, UK.

## Abstract

A registry including information about nearly 1,600 cases of retinoblastoma diagnosed in Britain has been created at the Childhood Cancer Research Group. Cases have been classified as 'old germ cell mutation', 'new germ cell mutation' or 'sporadic non-hereditary'. For a population-based group of 918 cases diagnosed between 1962 and 1985 we have calculated the proportions of unilateral/bilateral and hereditary/non-hereditary cases. Bilateral cases represent 40% of the total number over this period; the proportion known to be hereditary is 44%, a higher proportion than has been reported elsewhere. By following up selected groups of cases, an estimate has been made of the proportions of siblings of retinoblastoma patients and offspring of survivors from retinoblastoma who are themselves affected with the disease. Where there is no previous family history, the risk for siblings of retinoblastoma patients of developing the disease is approximately 2% if the disease in the affected child is bilateral and 1% if it is unilateral, assuming that there are no other siblings; if there are unaffected siblings the risks for subsequent children are lower. Children of patients with hereditary retinoblastoma have a one in two chance of carrying the germ cell mutation and for those who are carriers the probability of developing retinoblastoma is very close to the accepted figure of 90% if the parents have bilateral retinoblastoma but probably less if they have the unilateral form. For children of patients not known to be carriers, the probability of developing retinoblastoma is estimated to be about 1%, considerably lower than the previously accepted figure of about 5%. Retinoblastoma kindreds consist mainly of bilateral cases but there is evidence that some kindreds have a high proportion of unilateral cases. The ways in which these findings may be used in conjunction with modern techniques of molecular biology for prenatal and postnatal genetic counselling are discussed.


Br. J. Cancer (1992), 66, 211–219 © Macmillan Press Ltd., 1992

### Patterns of risk of hereditary retinoblastoma and applications to genetic counselling

G.J. Draper, B.M. Sanders, P.A. Brownbill & M.M. Hawkins

*Childhood Cancer Research Group, Department of Paediatrics, University of Oxford, Oxford, UK.*

**Summary** A registry including information about nearly 1,600 cases of retinoblastoma diagnosed in Britain has been created at the Childhood Cancer Research Group. Cases have been classified as 'old germ cell mutation', 'new germ cell mutation' or 'sporadic non-hereditary'. For a population-based group of 918 cases diagnosed between 1962 and 1985 we have calculated the proportions of unilateral/bilateral and hereditary/non-hereditary cases. Bilateral cases represent 40% of the total number over this period; the proportion known to be hereditary is 44%, a higher proportion than has been reported elsewhere. By following up selected groups of cases, an estimate has been made of the proportions of siblings of retinoblastoma patients and offspring of survivors from retinoblastoma who are themselves affected with the disease. Where there is no previous family history, the risk for siblings of retinoblastoma patients of developing the disease is approximately 2% if the disease in the affected child is bilateral and 1% if it is unilateral, assuming that there are no other siblings; if there are unaffected siblings the risks for subsequent children are lower. Children of patients with hereditary retinoblastoma have a one in two chance of carrying the germ cell mutation and for those who are carriers the probability of developing retinoblastoma is very close to the accepted figure of 90% if the parents have bilateral retinoblastoma but probably less if they have the unilateral form. For children of patients not known to be carriers, the probability of developing retinoblastoma is estimated to be about 1%, considerably lower than the previously accepted figure of about 5%. Retinoblastoma kindreds consist mainly of bilateral cases but there is evidence that some kindreds have a high proportion of unilateral cases. The ways in which these findings may be used in conjunction with modern techniques of molecular biology for prenatal and postnatal genetic counselling are discussed.

Although a rare tumour, comprising only about 3% of all childhood cancers in Western countries, retinoblastoma is of particular interest to geneticists and molecular biologists. It affects about one in 20,000 children, and occurs in a hereditary form in about 40% of cases. Previous studies have suggested that a predisposing germ line mutation is inherited from an affected parent in about 10% of cases and a new germinal mutation acquired in a further 30%. The remaining cases have sporadic unilateral retinoblastoma; a small proportion of these do in fact have a germinal mutation and are at risk for passing the disease to their children (Vogel, 1979).

The pattern of inheritance is that of a dominant gene, though the mutated retinoblastoma gene behaves as a recessive gene at the cellular level. The function of the wild type allele Rb+ at the retinoblastoma locus appears to be to maintain normal cellular growth control, i.e. it is a 'tumour suppressor gene'. Deletion or mutation of both alleles at this locus in a retinal cell can lead to retinoblastoma (Knudson, 1978; Murphree & Benedict, 1984; Dryja *et al.*, 1986; Friend *et al.*, 1986). The hereditary form of retinoblastoma, which occurs in families where there has been a germ cell mutation of the Rb gene, is incompletely penetrant; about 10% of carriers of the mutated gene do not develop retinoblastoma.

Non-hereditary retinoblastoma is caused by two mutations to a somatic cell, and the patient is only affected unilaterally.

Patients with the hereditary form of retinoblastoma who survive after treatment for the disease have a greatly increased risk of developing second non-ocular neoplasms. In adolescence, the risk is particularly high for osteosarcoma, and the increased risk for other neoplasms has been shown to continue in later life (Abramson *et al.*, 1984; Draper *et al.*, 1986; Sanders *et al.*, 1989).

Information about nearly 1,600 cases of retinoblastoma ascertained by the Childhood Cancer Research Group (CCRG) has been used to create a registry consisting of a computer database of linked files from which information can be abstracted to enable studies of selected groups of cases and families to be carried out. Various sets of data have been selected in order to study the risks that further cases of retinoblastoma may develop in families with affected children. In particular we have studied the risk that siblings may be affected after the appearance of retinoblastoma in one child in a family, and the risk for survivors from retinoblastoma that their children will develop the disease.

### Description of registry

### *Sources of ascertainment*

The Childhood Cancer Research Group has been notified of all cases of retinoblastoma registered through the National Cancer Registration Scheme in Britain from 1962 onwards. In addition, at certain centres of treatment for retinoblastoma, all patients treated in specified periods before 1962 have been ascertained. Death certificates for patients dying from retinoblastoma in England and Wales since 1953 have been received from the Office of Population Censuses and Surveys and those for Scotland from the General Register Office. Two interview studies were carried out on the above patients as part of the Oxford Survey of Childhood Cancers: one included children dying from retinoblastoma between 1953 and 1983 and the second included children registered with the disease between 1962 and 1971. Parents of the children were interviewed in these studies by medical staff from local authority health departments and by family doctors, and information was obtained relating to all aspects of the illness and to any known family history of retinoblastoma. A further series of interviews was carried out with the parents of children born between 1965 and 1985 attending Moorfields Hospital and St Bartholomew's Hospital in London for treatment or follow up after treatment for retinoblastoma. Complete pregnancy histories for the parents were obtained in the course of these three interview studies. Surviving patients have been followed up through hospital consultants and family doctors and by 'flagging' at the National Health Service Central Registers (NHSCR): for patients flagged in this way, cancer registrations for any subsequent

Correspondence: G.J. Draper, Childhood Cancer Research Group, 57 Woodstock Road, Oxford OX2 6HJ, UK.
Received 17 June 1991; and in revised form 13 February 1992.

tumours and death certificates are received routinely from the NHSCR.

In addition to the above groups of patients, all relatives of index cases known to have had retinoblastoma or, by inspection of the pedigree, discovered to have been unaffected carriers of the disease have been included in the registry. Currently, 137 families with more than one case of retinoblastoma are known to us. One family is known to include 19 patients with retinoblastoma.

*Classification of cases*

We have used the following criteria to divide all cases into three groups.

(i) Cases with a known family history of the disease either in a previous generation or in a collateral family line or with affected siblings have inherited a germ cell mutation probably from at least two generations back. We have called these *old germ cell mutation cases.*

(ii) Cases which are bilateral but have no previous known family history are called *new germ cell mutation cases*; also in this category we have placed those unilateral cases which represent the first appearance of retinoblastoma in a family if there is subsequently an affected child. We have assumed here that the most likely explanation for the occurrence of such cases is a germ cell mutation in an unaffected parent of the index case.

(iii) The remaining unilateral cases with no known family history of retinoblastoma are referred to as *sporadic non-hereditary cases.* A small proportion of this last group may subsequently be discovered to be hereditary because a child of such a patient or some other family member develops retinoblastoma.

*Descriptive epidemiology and family studies*

The following sections describe a series of three studies carried out using information included in the registry. In the next section we summarise information on a series of 918 cases of retinoblastoma diagnosed between 1962 and 1985. These cases have been categorised by laterality and by whether or not there was a known family history of retinoblastoma. Mean and median ages at diagnosis have been calculated for the separate groups. In the Section on Sibships of retinoblastoma cases, information about affected and unaffected siblings of retinoblastoma patients obtained during interviews with their parents has been analysed to estimate the risk that the sibling of an affected proband will develop retinoblastoma. In the Section on Offspring of retinoblastoma cases, pregnancy histories of survivors from retinoblastoma obtained from questionnaires completed by their family doctors have been analysed to estimate the risk that offspring of these survivors will themselves develop retinoblastoma.

### Incidence and age distributions for different types of retinoblastoma

Table I includes 918 patients diagnosed between 1962 and 1985, the 24 years of national data for which we have almost complete ascertainment. Cases are classified according to their laterality and whether the retinoblastoma is hereditary or non-hereditary. Altogether 364 patients, 40% of all cases, were affected bilaterally. A total of 151 cases (16%) are known to have inherited a mutation of the Rb gene because there is a family history, and 255 cases (28%) with no known family history are apparently caused by a new germ cell mutation. Thus a total of 406 cases (44%) are known to be hereditary; the remaining 512 cases (56%) are so far considered to be non-hereditary.

Table II shows the mean and median ages and age distribution at diagnosis for the cases in Table I. As has been reported in previous studies (Leelawongs & Regan, 1968; Matsunaga & Ogyu, 1976; Sanders *et al.*, 1988) bilateral cases tend to be diagnosed at a much younger age than unilateral cases. For patients with an old germ cell mutation, that is those with a family history, the mean age at diagnosis for bilateral cases was 7.2 months, half of the cases being diagnosed by 5 months; for unilateral cases in this group the mean age at diagnosis was 20.3 months. The mean age at diagnosis for sporadic unilateral cases was 29.5 months, half of the cases being diagnosed by 26 months.

### Sibships of retinoblastoma cases

*Methods*

Information about 766 families of retinoblastoma patients, ascertained during interviews with parents in the three interview studies described above, was included in this study. A complete pregnancy history for each family was obtained up to the date the parents were interviewed. If any of the siblings had developed retinoblastoma the laterality and date of diagnosis were noted.

*Results*

The total numbers of liveborn children in the above families, and the numbers affected by retinoblastoma are shown in Table III. In this table and subsequent analyses the category 'previous family history' excludes those families where there are sib pairs but no other affected relatives.

One of the objectives of the present paper is to determine, in families where there was a child with retinoblastoma, the risk that other children in the family would develop retinoblastoma, and thus to produce information useful for genetic counselling, based on a large number of families. Only families with at least one liveborn child in addition to the index child have been included in these analyses.

A total of 622 families with 1,905 live born children were included in the analysis. In 34 of these families there was more than one child with retinoblastoma: one family included three and one family four affected children. Two families included monozygotic twins, both of whom developed retinoblastoma; for the present analysis, each pair has been counted as a single case of retinoblastoma.

The information from these 622 families has been used to calculate the risks of retinoblastoma among the siblings of affected children classified into groups according to whether

**Table I** Numbers of retinoblastoma cases diagnosed between 1962 and 1985 subdivided by laterality and by type of retinoblastoma

| *Year of diagnosis* | *Old germ cell mutation* | | *New germ cell mutation* | | *Non-hereditary* | |
|---|---|---|---|---|---|---|
| | *Bilateral* | *Unilateral* | *Bilateral* | *Unilateral* | *Unilateral* | *Total* |
| 1962–1963 | 11 | 6 | 23 | 0 | 35 | 75 |
| 1964–1968 | 33 | 6 | 58 | 0 | 114 | 211 |
| 1969–1973 | 14 | 10 | 56 | 0 | 127 | 207 |
| 1974–1978 | 17 | 10 | 52 | 0 | 117 | 196 |
| 1979–1983 | 24 | 7 | 43 | 0 | 83 | 157 |
| 1984–1985 | 10 | 3 | 23 | 0 | 36 | 72 |
| Total | 109 | 42 | 255 | 0 | 512 | 918 |

**Table II** Age distribution and mean and median ages at first diagnosis for retinoblastoma cases diagnosed between 1962 and 1985, subdivided by laterality and by type of retinoblastoma

| *Age at diagnosis (months)* | *Old germ cell mutation* | | *New germ cell mutation* | | *Non-hereditary* | |
|---|---|---|---|---|---|---|
| | *Bilateral* | *Unilateral* | *Bilateral* | *Unilateral* | *Unilateral* | *Total* |
| 0–5 | 64 | 12 | 61 | 0 | 28 | 165 |
| 6–11 | 23 | 4 | 70 | 0 | 63 | 160 |
| 12–17 | 11 | 9 | 51 | 0 | 55 | 126 |
| 18–23 | 5 | 3 | 31 | 0 | 75 | 114 |
| 24–29 | 3 | 3 | 19 | 0 | 78 | 103 |
| 30–35 | 0 | 2 | 13 | 0 | 62 | 77 |
| 36–41 | 0 | 2 | 5 | 0 | 46 | 53 |
| 42–47 | 2 | 1 | 1 | 0 | 30 | 34 |
| 48–53 | 0 | 1 | 1 | 0 | 28 | 30 |
| 54–59 | 0 | 1 | 1 | 0 | 11 | 13 |
| 60–119 | 0 | 3 | 1 | 0 | 34 | 38 |
| 120 + | 0 | 0 | 1 | 0 | 2 | 3 |
| Total | 108 | 41 | 255 | 0 | 512 | 916 |
| Mean age at diagnosis (months) | 7.2 | 20.3 | 14.0 | – | 29.5 | 22.1 |
| Median age at diagnosis (months) | 5 | 15 | 11 | – | 26 | 18 |

Two children with hereditary retinoblastoma have been excluded from this table because they presented with regressed tumours.

**Table III** Numbers of liveborn children and cases of retinoblastoma in families included in the sibship study

| | *Hereditary* | | | |
|---|---|---|---|---|
| | *Previous family history* | *No previous family history* | *Non-hereditary* | *Total* |
| Children with bilateral retinoblastoma | 110 | 274 | 0 | 384 |
| Children with unilateral retinoblastoma | 31 | 6 | 382 | 419 |
| Unaffected siblings | 81 | 467 | 698 | 1246 |
| Total number of live births | 222 | 747 | 1080 | 2049 |
| Live births included in the analysis | 186 | 696 | 1023 | 1905 |
| Number of families | 109 | 275 | 382 | 766 |
| Number of families included in the analysis | 73 | 224 | 325 | 622 |

they had unilateral or bilateral retinoblastoma and to whether there was a family history of the disease in either a previous generation or a collateral line; the method of analysis adopted depends on whether there is known to be such a mutation.

*(i) Families where there is a previous family history* For these families the risk of retinoblastoma among the siblings (excluding monozygous co-twins) is estimated (separately for bilateral and unilateral probands) using standard actuarial methods taking into account the length of follow-up, i.e. the age of the sibling at the time of the interview with the family or at the development of retinoblastoma or death from some other cause. Families in which there is more than one independently ascertained child with retinoblastoma (other than monozygous twins) are included in the analysis more than once, each child with an independent ascertainment appearing in turn as the index child. This point is discussed in the Appendix, Section A2.

The numbers of cases and the results of these calculations are shown in Table IV which gives estimates of the probability of retinoblastoma developing among siblings, subdivided according to whether the proband is bilaterally or unilaterally affected (The standard errors in this table may be underestimated; see Appendix, Section A2.) The probability shown is the chance of a sibling developing the disease by age 6 years. It is rare for retinoblastoma to develop after 6 years, and in the present series no sibling developed retinoblastoma after the age of four; thus these are effectively the risks of ever developing retinoblastoma.

For siblings of bilaterally affected cases the probability of retinoblastoma developing by age 6 years is 44.8%, which is the figure normally quoted for the probability of retinoblastoma in families with the hereditary form of the disease (45%); for siblings of unilaterally affected cases the corresponding figure is 30%. The majority of cases have developed by age 1 year. There is strong evidence from these data that bilateral probands nearly always have bilateral siblings (47 out of 51 affected siblings being bilaterally affected) whereas unilateral probands have unilateral siblings rather more frequently than they have bilateral ones (seven out of 11).

*(ii) Families where there is not a previous family history* For these families the estimation procedure has to allow for the fact that there are in fact two groups, those with a previously unrecognised germ cell mutation or gonadal mosaicism, and those where the retinoblastoma in the offspring is due to a somatic mutation or a mutation in just one parental germ cell. In the first group of families it is quite likely that a second child will be born with retinoblastoma whereas this is

**Table IV** Estimated probabilities of developing retinoblastoma for sibs of probands where there is a previous family history

| Type of proband | No. of sibs[a] at risk | No. of affected sibs[a] Bilateral | Unilateral | Total | Estimated % of sibs developing retinoblastoma by age 6 years (standard error) |
|---|---|---|---|---|---|
| Bilateral | 120 | 47 | 4 | 51 | 44.8 (5.3) |
| Unilateral | 38 | 4 | 7 | 11 | 30.0 (9.0) |

[a]In families with more than one independently ascertained proband, sibs are counted more than once.

extremely unlikely for the second group.

The probability of a subsequent child being affected has to be estimated (separately for bilateral and unilateral probands) taking into account the number of unaffected siblings in the family. (It is assumed that there is only one affected child; after a second affected sibling the family can be assigned to the old germ cell mutation category).

The method of calculation is explained in Appendix Section A3, and the results presented in Table V. For simplicity we have given estimates, separately for bilateral and unilateral probands, only for a second and fourth child born into a family, i.e. for the case where the affected child is the only child in the family and the case where there are also two unaffected siblings. As can be seen from Table V the estimated risk if there is just one affected case and no other children in the family is about 2% for siblings of bilateral cases and about 1% for siblings of unilateral cases. The estimated risks are highest if the affected child is the only child in the family and decrease as the number of unaffected children increases: for the second case considered, i.e. where in addition to the one affected child there are two unaffected siblings, the estimated risks (i.e. for the fourth child in the family) become about 0.6% if the affected child's retinoblastoma is bilateral and about 0.5% if it is unilateral. The probabilities for other family sizes can be easily calculated from that for families of size one as shown in Appendix Section A3.

A measure of the precision of the estimates may be obtained by regarding the number of sibships with a second case as a Poisson variable. In this study there were two such sibships for both the bilateral and the unilateral probands. The 95% confidence limits for a Poisson mean when the observed value is 2 are 0.24 and 7.2 and thus 95% lower and upper confidence limits for each of the risk estimates given above can be obtained by multiplying them by 0.12 and 3.6 respectively. For instance in families where there is just one affected child (and no previous history of a germ cell mutation) the lower and upper confidence limits for the risk that the second child will be affected are about 0.2% and 7% if the first child is a bilateral case, and 0.1% and 4% if it is unilateral.

*(iii) Other findings in the sibships* It is interesting to note that, in addition to the occurrence of retinoblastoma among the siblings of these index children, two of the siblings of sporadic cases of retinoblastoma developed non-ocular cancer. One child was diagnosed with osteosarcoma aged 14 years, and the second developed acute lymphoblastic leukaemia before he was two. In view of findings from many previous reports about the relationship of the retinoblastoma gene to other cancers (e.g. Sanders *et al.*, 1989) it seems likely that the first, and perhaps both, of these are examples of cases in which the loss of the retinoblastoma gene does not lead to retinoblastoma.

The numbers of miscarriages and stillbirths in these families were also studied to see whether there was any excess risk of these events in the different categories of family. There is some evidence that the mothers of children with malignant disease tend to have a greater number of miscarriages than mothers in the general population. They are also known to have a slightly increased chance of bearing another child with cancer (Draper *et al.*, 1977), and there are known associations between childhood cancers and some congenital abnormalities and genetic diseases. If, as seems likely, the fetus in another pregnancy has an increased chance of miscarrying because of one of these conditions, this would explain the observed association. In retinoblastoma families it is possible that parents carrying a germ cell mutation might also have an increased history of miscarriages, while parents of non-hereditary cases should have no increased risk. In the present series there is no evidence of any difference in the risk of miscarriage or stillbirth between the groups.

### Offspring of retinoblastoma cases

### *Methods*

A previous study of offspring of 1,348 survivors from cancer born in 1962 or earlier included 263 retinoblastoma patients (Hawkins *et al.*, 1989). Questionnaires were sent to their family doctors requesting information about the present health of these patients, whether they had any liveborn children, stillbirths, miscarriages, or terminations of pregnancy and whether any of the children had developed retinoblastoma. One hundred and fifty-seven children were born to 89 of these patients. This study has now been extended and up-dated, bringing the numbers for whom current information about retinoblastoma survivors and their families has been obtained up to 316. A further 36 questionnaires sent to family doctors were not returned: there was thus an overall 90% positive response rate for the study.

### *Results*

The numbers of known pregnancies, miscarriages, stillbirths and liveborn children among female survivors and partners of male survivors from retinoblastoma have been calculated

**Table V** Estimated probabilities of developing retinoblastoma for sibs of probands where there is not known to be a previous family history

| Type of proband | No. of sibs at risk | No. of affected sibs Bilateral | Unilateral | Total | Estimated % of sibs developing retinoblastoma if there is just one affected sib and (a) none unaffected | (b) two unaffected |
|---|---|---|---|---|---|---|
| Bilateral | 159 | 2 | 0 | 2 | 2.1[a] | 0.6[a] |
| Unilateral | 271 | 1 | 1 | 2 | 1.1[b] | 0.5[b] |

[a]Assuming a 90% penetrance in the subgroup of these families where there is a germ cell mutation.
[b]Assuming a 60% penetrance in the subgroup of these families where there is a germ cell mutation.

separately for parents classified as being old germ cell mutation, new germ cell mutation and non-hereditary cases. A total of 197 survivors were not known to have any liveborn children. (For 31 male survivors the family doctors stated that they were not certain they would have known of any children).

A total of 119 survivors had at least one liveborn child. The families of 44 patients (15 males, 29 females) with hereditary retinoblastoma included 78 children born before the interview date, and 32 of them developed retinoblastoma: 26 children were affected bilaterally and six children unilaterally. There were two offspring with retinoblastoma among the six children born to survivors of hereditary unilateral retinoblastoma; these are included in the analysis, which is based mainly on children born to survivors with bilateral retinoblastoma. Three children (unaffected by retinoblastoma) who died within a few days of birth have been excluded from the analysis. The probabilities of retinoblastoma occurring in the offspring of survivors from hereditary retinoblastoma by age 1 and 6 years have been calculated from these data using the method described in the statistical appendix, Section A1, and the results are shown in Table VI. (The standard errors may be somewhat overestimated; see Appendix A1.)

The probability of retinoblastoma occurring in children of survivors of hereditary retinoblastoma – 43.5% by age 6 years – is, as with the sibship study, very close to the usually quoted figure of 45%. We are also aware of five survivors from bilateral retinoblastoma included in this survey who had affected children and who have been excluded from the analysis because details of these patients were not obtained from their family doctors and information about their other offspring was therefore not available. Of these, one questionnaire was not returned, two survivors had children who developed retinoblastoma after the end of study date, and for two survivors the family doctor did not know about the affected children. These cases of retinoblastoma were notified to us through cancer registries.

In addition to the offspring with retinoblastoma one child of a patient with hereditary retinoblastoma developed a malignant testicular teratoma.

It is particularly important to try to make some estimate of the risk of retinoblastoma among the offspring of survivors of *sporadic unilateral* retinoblastoma. Among 75 survivors (34 males, 41 females) for whom questionnaires were returned and who had offspring, 139 children were born before the interview date and there were no affected cases among these children. Among the 36 survivors included in the questionnaire study for whom no response was received, 24 were sporadic unilateral cases, and it is possible that the information on numbers of pregnancies and offspring was incorrect in a further 13 such cases where the family doctors may not have had complete information. Thus for 37 patients, 22 males and 15 females, we do not know about the size of their families, but it is reasonable to assume that we would know about any affected offspring from cancer registration records or other sources of ascertainment; we do in fact know through cancer registration of one child with retinoblastoma born to one of these female patients for whom no reply was received from the family doctor. There were in total 165 completed questionnaires for the sporadic unilateral cases. Assuming that the family sizes were similar for those from whom no information is available we have estimated that the numbers of families with 0, 1, 2, 3 . . . children were as shown in Table VII. On the basis of these assumptions, and assuming that all offspring are followed up to the age by which any retinoblastomas would have appeared, we can estimate the probability that a unilateral case with no family history is in fact carrying a germ cell mutation, using the method of maximum likelihood estimation as described in the Appendix, Section A4. Assuming a penetrance of 90% the estimated probability is about 1.7% with a standard error also of 1.7%. If however, as suggested by Matsunaga (1978) and der Kinderen (1987) the penetrance is lower for the offspring of unilateral cases then the estimate of the proportion of hereditary cases is higher. If we assume a penetrance of 60% for the offspring of unilateral cases (in line with the estimated risk of 30% to siblings of such cases found in Table IV) this probability becomes 2.3% with a standard error of 2.3%. In either case the estimated risk of retinoblastoma among the children of such patients is about 0.7% –0.8%, with a standard error of the same magnitude.

**Table VI** Estimated probability of developing retinoblastoma for offspring of parents with hereditary retinoblastoma

| *No. of survivors with offspring* | *No. of offspring* | *No. of affected offspring* Bilateral | *Unilateral* | *Total* | *% of offspring developing retinoblastoma by stated age (standard error)* 1 year | *6 years* |
|---|---|---|---|---|---|---|
| 44 | 75[a] | 26 | 6 | 32 | 38.7 (5.6) | 43.5 (7.0) |

[a]This total excludes three children (unaffected by retinoblastoma) who died within a few days of birth.

**Table VII** Estimated family size distribution for 202 survivors of sporadic unilateral retinoblastoma

| *No. of liveborn offspring* | *Survivors for whom GP was able to provide information* Males | *Females* | *Estimate [a]for cases where GP could not provide information* Males | *Females* | *Estimated family size distribution for complete group* Males | *Females* | *Both* |
|---|---|---|---|---|---|---|---|
| 0 | 27 | 63 | 10 | 8 | 37 | 71 | 108 |
| 1 | 15 | 14 | 5 | 3[b] | 20 | 17[b] | 37[b] |
| 2 | 13 | 20 | 5 | 3 | 18 | 23 | 41 |
| 3 | 5 | 4 | 2 | 1 | 7 | 5 | 12 |
| 4 | 1 | 2 | 0 | 0 | 1 | 2 | 3 |
| 5 | 0 | 1 | 0 | 0 | 0 | 1 | 1 |
| Total | 61 | 104 | 22 | 15 | 83 | 119 | 202 |

[a]The numbers in this column are calculated by assuming that the 22 male patients for whom family size information was not obtained from the general practitioner had family sizes in similar proportions to those male patients for whom information *was* provided. Similarly for the female patients, the one case of retinoblastoma in a child born to such a mother being added separately and not included in these calculations. [b]Includes family with one child with retinoblastoma born to a survivor classified as having sporadic unilateral retinoblastoma.

As with the study of other pregnancies among parents of children with retinoblastoma we thought it possible that survivors of retinoblastoma might have an increased miscarriage or stillbirth rate. However there was no evidence of such an increase.

### Discussion

A considerable range of figures has been published concerning the proportions of unilateral/bilateral cases and hereditary/non-hereditary cases of retinoblastoma. On the basis of the large numbers of cases of retinoblastoma presented in this paper diagnosed between 1962 and 1985, a period for which we believe we have good ascertainment, and where the cases have been followed up through family doctors and clinicians to verify the diagnosis and family history, we suggest that the distribution of cases in Britain is as follows.

Bilateral cases represent 40% of the total number; of these 28% have a family history at the time of diagnosis. Of the 60% of cases which are affected unilaterally, 7% have a previous family history. In all, 15% of cases have a family history of retinoblastoma at the time of diagnosis. Our suggested proportions of 44% hereditary and 56% non-hereditary cases may be subsequently affected by further cases of retinoblastoma appearing in the families of those who on present information are placed in the sporadic non-hereditary category.

The proportion of bilaterally affected cases in this study (40%) is higher than that reported in many other studies. Some studies are subject to selection bias and not too much reliance should be placed on the proportions quoted. However, the Surveillance, Epidemiology and End Results (SEER) study of children's cancer in the United States includes 220 cases of retinoblastoma ascertained from nine population based registries, and the proportion of bilateral cases quoted in this study was only 25% (Tamboli et al., 1990). It is possible that some of the cases originally ascertained as unilateral in this study later developed tumours in the other eye. Among 550 cases ascertained in the Netherlands, 31% were found to be bilateral (Schappert-Kimmijser et al., 1966), and in a study of 899 cases of retinoblastoma in France, 34% were bilateral (Bonaiti-Pellie, 1976).

For comparison with our figure of 44% for the proportion of all hereditary cases, Der Kinderen et al. (1988) found that 36% of the total of 403 cases in the Netherlands retinoblastoma registry diagnosed between 1945 and 1970 were hereditary. A subgroup of 598 patients in Bonaiti-Pellie's study where a complete family history was obtained showed a proportion of 40% of patients to have hereditary retinoblastoma.

It has been suggested that the proportion of hereditary cases in the population will increase with improved survival (Vogel, 1979). We have not observed this in our figures (see Table I), but are not able to obtain reliable population based data for the years before 1962.

### *Age at diagnosis*

The data on average age at diagnosis in Table II confirm and extend those from previous studies. It is well recognised that patients with the hereditary form of retinoblastoma tend to be diagnosed earlier than those with the non-hereditary form and that bilateral cases tend to be diagnosed earlier than unilateral ones; since bilateral cases are hereditary and unilateral cases mainly non-hereditary these two comparisons to some extent overlap. In Table II we have attempted to separate the two effects. The first two columns compare hereditary unilateral and bilateral cases. A possible explanation of the difference between bilateral and unilateral cases is that the occurrence of bilateral disease may be an indication that the individual or family is more susceptible, or more exposed to mutagenic agents, than those where the disease is unilateral; retinoblastoma would be expected to develop earlier in the former. For hereditary cases without a family history (new germ cell mutations) the bilateral cases are diagnosed later than those with a family history; this may simply be a consequence of the fact that these patients would not have had the regular eye examination that those with a family history have.

Non-hereditary unilateral cases are on average diagnosed later than any of the other groups. This is well recognised and can be predicted as a consequence of the hypothesis that such cases have to accumulate two somatic mutations rather than one before retinoblastoma develops (Knudson, 1978).

### *Risks to siblings*

When parents are known to be carriers of the retinoblastoma gene, the risk to their children of inheriting the mutated gene is 50%. The risk that the gene is expressed as retinoblastoma is commonly accepted to be 90%, though this may be lower if the parent is unilaterally affected. Using life-table methods as described in the Section 'Sibships of retinoblastoma cases: Results (i)', to allow for varying periods of follow-up, the estimated risk for the siblings of bilaterally affected cases in such families is 44.8%, corresponding to a penetrance of 89.6%. This compares with the generally accepted estimate of 90%. The life-table estimate of the risk of retinoblastoma for the siblings of unilaterally affected children in such families is 30%, giving an estimated penetrance of 60%.

When a new case of retinoblastoma appears in a family with no previous family history of the disease, parents are naturally anxious to know the risk that a subsequent child might be affected. The difficulty in estimating this risk arises from the different ways in which the retinoblastoma may have arisen. It may be the result of somatic mutations in the affected child or of a new mutation affecting a single parental germ cell; there is no increased risk to other siblings in either of these cases. Alternatively it may have been the result of an unrecognised old germ cell mutation or of gonadal mosaicism in a parent. The risk to the siblings of sporadic cases is an average of the separate risks for these various types of family. This average risk has been assessed by Vogel (1979) as 6% after the birth of a bilateral sporadic case and 1% after the birth of a unilateral sporadic case. From our study of families where, apart from the index child, there was no family history of retinoblastoma, we conclude that if there is just one affected child and no unaffected children in the family the risk that the next child will be affected is 2% for siblings of a bilaterally affected child and about 1% for siblings of a unilaterally affected child. As explained in the Section 'Sibships of retinoblastoma cases: Results (ii)' and 'Appendix A3', these risks are lower if there are also some unaffected children in the family. This latter point is referred to but not discussed by Vogel whose estimates appear to be averages taken over all sibship sizes. Taking this into account, our estimate for siblings of sporadic unilateral cases is rather lower than Vogel's; allowing for the degree of uncertainty in the estimates they are consistent with each other. Our estimate for siblings of sporadic bilateral cases is considerably lower than Vogel's, particularly since it again relates to a sibling of an only, affected, child (the type of family for which the estimated risk to a subsequent child is highest) while Vogel's is an average for different family sizes.

### *Risks to offspring*

In this part of the study, the likelihood of passing on the mutated retinoblastoma gene to their children is assumed to be the same for both old germ cell and new germ cell (sporadic bilateral) cases of retinoblastoma. Among 75 children born to the 44 survivors with the hereditary form of retinoblastoma who had liveborn children 32 developed retinoblastoma. Using life-table methods it can be estimated that the proportion developing retinoblastoma by age 6 years is 43.5%, giving a penetrance of 87%. Again this is very close to the usual assumption of a 90% penetrance.

The analysis in the Section 'Offspring of retinoblastoma cases: Results' and 'Appendix Section A4', suggests that for children born to survivors from unilateral sporadic retinoblastoma the risk is about 1%, the proportion of unilateral sporadic cases who are in fact carrying the retinoblastoma gene being estimated as about 2%. This value updates the previous estimate, given in Hawkins *et al.* (1989), which was based on an earlier analysis of a subset of the cases in the present paper. The estimated risk to subsequent children after the birth of an affected child is of course the same as that for other parents with hereditary retinoblastoma. Again, for each *unaffected* child born to a possible carrier the estimated probability that subsequent children will be affected *decreases*.

For unilateral sporadic cases, Vogel (1979) has suggested that between 10% and 12% of such cases are caused by germ cell mutations and therefore that about 5% of their children may be affected with retinoblastoma. Vogel bases his estimate, which is the one nearly always quoted for genetic counselling, on the joint results from seven separate studies, the largest study (Schappert-Kimmijser *et al.*, 1966), giving a particularly high rate of affected children in the families of survivors from unilateral sporadic retinoblastoma. If the selection of cases included in some of these seven studies was biased this could lead to an overestimate of the risk: such bias could arise for instance if unilateral sporadic probands were included in a series after being ascertained through an affected offspring. Again, if inadequate family histories were obtained cases could be wrongly classified as sporadic.

*Non-ocular tumours*

It is well known that survivors of hereditary retinoblastoma have a greatly increased risk of developing a variety of other tumours (Draper *et al.*, 1986; Sanders *et al.*, 1989) and it has been suggested that there may be an increased risk even among unaffected family members. In the present study we found two cases of childhood cancer, one osteosarcoma and one acute lymphoblastic leukaemia among 1,246 unaffected siblings in retinoblastoma families, both occurring among the 698 siblings of sporadic non-hereditary cases. This represents a rather higher incidence than that found in the general population but cannot necessarily be considered as evidence of an increased risk in non-carriers, particularly as it seems reasonable in view of the well known association between retinoblastoma and osteosarcoma to speculate that the case of osteosarcoma might have arisen in a child with unexpressed retinoblastoma.

In the study of offspring, one of the 217 children identified in the follow-up studies developed a testicular teratoma. This, together with a case of a sib of a patient with sporadic unilateral retinoblastoma who developed a teratoma of the testis (included in the registry but not in this study) raises the question of whether there is a real association between these two conditions. We have also noted that a child born to a survivor from sporadic unilateral retinoblastoma died from neuroblastoma. We do not know of any previous reference to an association between these two neoplasms and this may well be a chance finding.

*Genetic counselling*

The results of this paper are obviously relevant to problems of genetic counselling. For patients with hereditary retinoblastoma our findings agree with the generally quoted risk of retinoblastoma to their offspring of 45% – arising from a 50% risk of inheriting the retinoblastoma gene, together with a penetrance of 90%. The risks to various types of relative can be calculated in the same way as for any dominant gene; see for example the discussion in Harper (1988) Chapter 2.

The risks for patients with sporadic unilateral retinoblastoma and their relatives appear to be much smaller than the estimates quoted in earlier papers. As explained above most of these estimates seem to be based on Vogel's (1979) review and there is some uncertainty about the selection of cases in the series on which he bases his estimates. Our estimate of the probability that a unilateral sporadic case is in fact a gene carrier is about 2%, perhaps higher if the case has no siblings, and decreasing as the number of unaffected siblings increases. The probability that the gene will be transmitted to the children of such patients is about 1%. The estimated risk for a sibling of a unilateral sporadic case, when there are no other siblings, is similar. Again this risk decreases as the number of unaffected siblings increases. For siblings of patients with bilateral retinoblastoma our estimate of the risk is about 2%, less if there are already unaffected siblings; this is lower than that of Vogel, which was based on the study by Briard-Guillemot *et al.* (1974), of about 6%. It is not clear whether the substantial difference between these estimates is due to the fact that both are rather imprecise or whether in the Briard-Guillemot study some family histories were missed and the cases wrongly classified as sporadic.

Genetic counselling for other relatives of these patients can again be based on standard methods for such diseases (Harper, 1988, Chapter 2).

With recent developments in molecular genetics it is of course possible, in certain situations, to make considerably better risk estimates:

(i) Where at least two family members are affected it is possible, using restriction fragment length polymorphisms (RFLPs) to apply standard methods of genetic linkage analysis to identify gene carriers with a high degree of certainty; the range of RFLPs now available mean that the great majority of families will be informative using this method (Wiggs *et al.*, 1988). These techniques have been applied both prenatally and postnatally (Onadim *et al.*, 1990).

(ii) Even for sporadic cases it may be possible to distinguish between hereditary and non-hereditary cases using the approach described by Yandell *et al.* (1989) which involves the direct identification of point mutations in the retinoblastoma gene and compares tumour cells with constitutional cells.

Cowell (1991) in a review of the molecular genetics of retinoblastoma stated that identification of all gene carriers in retinoblastoma families will soon be possible. This would mean that the frequent ophthalmological examinations under anaesthetic of all children of affected parents and other relatives of retinoblastoma patients would no longer be necessary, and clinical resources could be concentrated on patients who are carriers.

This paper is based on information provided by cancer registries, hospital consultants and family doctors in Britain. We would like to thank them, and are especially grateful to the parents of children included in this study who agreed to be interviewed and to give information about their families; the data from some of these interviews was kindly made available to us by the staff of the Oxford Survey of Childhood Cancer in Birmingham. The Office of Population Censuses and Surveys, the Information and Statistics Division of the Common Services Agency of the Scottish Health Service, the Registrar General for Scotland and Regional Cancer Registries all provided notifications of retinoblastoma cases, and we are grateful to them. We would also like to thank Dr M. Jay and Dr J.E. Kingston of Moorfields and St Bartholomew's Hospitals for information provided about patients and their families. We are grateful to Mrs K. Bunch, Mrs E. Mowat, Mrs E. Roberts and Mr M. Loach for help with setting up the database, collection of data and computer calculations.

We are grateful to one of the referees for suggestions on the analysis of the risks to siblings and for pointing out that our results had implications for the estimation of the proportions of non-penetrant cases of hereditary retinoblastoma.

The Childhood Cancer Research Group is supported by the Department of Health and the Scottish Home and and Health Department. The work of collecting family data was also supported in part by the Cancer Research Campaign.

### APPENDIX: STATISTICAL METHODS

In Section A1, A2, A3 we give details of the statistical methods used to estimate the risks to siblings of affected cases and to offspring of parents known to have the hereditary form of retinoblastoma. In Section A4 we explain the method used to estimate the proportion of hereditary cases among sporadic unilateral cases, on the basis of the number of cases of retinoblastoma observed in their offspring

### A1 Risks to offspring of hereditary cases

For simplicity we discuss first the estimation of the risk for offspring of hereditary cases. Virtually all cases of retinoblastoma become apparent by age 10 years; thus if all offspring were followed up to age 10 the proportion of affected cases would be simply the ratio of affected cases to total offspring. Since in practice the length of follow-up varies it is necessary to use actuarial, or life-table, methods in estimating the risk (see e.g. Peto *et al.*, 1976; 1977). This method is routinely used in clinical trials where patients are followed-up for differing lengths of time and where the length of survival to death or relapse is allowed for in the analysis. The essence of the method is that if we are interested in, say, mortality 5 years after treatment we cannot classify patients who have only been followed up for 3 years and are alive at that time; but they do, for the first 3 years, contribute to the denominator of those at risk for the first 3 years, and therefore must be taken into account in calculating the risk up to that point since this affects the risk at 5 years. Similarly, in the present analysis, offspring known to be alive at 3 years (but then lost to follow-up) contribute to the denominators of persons at risk up to that age, but not thereafter. In Table VI we give the actuarial estimates of the proportions of offspring of hereditary cases who developed retinoblastoma by age 1 year and by age 6 years. (The estimates of standard errors in this table are the conservative ones suggested by Peto *et al.*, 1977, i.e. they are likely to be overestimated.) Since, as shown in Table II, about 99% of affected hereditary cases develop the disease by age 5, the risk by age 6 is almost equivalent to the total risk.

### A2 Risks to siblings of affected cases in families with a previous family history

For the risks to siblings in these families it is necessary to take into account the fact that in some of the families with more than one affected child, the sibship was ascertained independently through two or more of these affected children. (This point is discussed in, for example, Cavalli-Sforza & Bodmer, 1971). If, for instance, the family contains two independently ascertained affected siblings A and B, and two unaffected siblings C and D the family contributes twice to the analysis as follows: first, regarded as siblings of A there are three siblings, B, C and D at risk, one of whom, B is affected; secondly, regarded as siblings of B, sib A is affected, C and D are unaffected. With this interpretation of the at-risk population the analysis proceeds in the same way as that for the offspring described above. The standard errors calculated from the life-table analysis (though again calculated according to the conservative method which overestimates the error of the usual life-table method) do not take the double ascertainments into account and this will lead to an underestimation. By analogy with the situation where all cases are identified during the period of follow-up, i.e. where actuarial methods are not necessary, it seems likely that the standard error is not more than 40% greater than the value quoted here (Cavalli-Sforza & Bodmer, pp. 856–7).

### A3 Risk to siblings of affected cases in families where there is not known to be a previous family history

As explained in the Section 'Sibships of retinoblastoma cases: Results (ii)', the estimation of the risk to siblings in these families is complicated by the fact that the families are actually a mixture of types – those where there is an old germ cell mutation (or gonadal mosaicism) and those where there is not. The affected children in the latter families may have retinoblastoma following a first mutation which affected either a parental germ cell or a somatic cell in the child: in either case there is no increased risk for sibs. If there are two affected children the recurrence risk for further children is probably the same as for families with known heritable retinoblastoma. On the other hand, for families with only one affected child the estimated recurrence risk depends on the number of unaffected children in the family: the estimates required for genetic counselling must take this into account. The risks depend on the proportions of families with unrecognised old germ cell mutations among those where one child has retinoblastoma. For genetic counselling, we need to estimate the probabilities of a second case occurring in families of specified sizes, given that there is one child affected and that the remainder are unaffected. We assume that these probabilities may depend on whether the affected child has bilateral or unilateral retinoblastoma. For instance, we require for a family of size s, including one child with bilateral retinoblastoma, and no family history, an estimate of the risk that the next child will be affected. Such probabilities could in principle be estimated empirically from the data on families of different size, but in practice the numbers of cases available are far too small for this approach; the method described here makes use of all the data to estimate the probability, $x_1$, that for a family with one sporadic case and no unaffected cases the family is carrying an old germ cell mutation (in which we include the possibility of gonadal mosaicism). The other probabilities of interest can be derived from $x_1$. For a family with two children of whom one is a sporadic case and one is unaffected let $x_2$ be the probability that there is an old germ cell mutation. In general denote this probability by $x_s$ if one of a family of s children is affected. Let p be the probability of an affected case occurring if the family is carrying an old germ cell mutation. On the basis of the results given in Table IV we assume that, for such families, if there is already an affected case, $p = 0.45$ if the case is bilateral and 0.3 if it is unilateral; let $q = 1 - p$.

Obviously, for a family with one out of s children affected the probability that there is *not* an old germ cell mutation is $1 - x_s$. In this case the probability that the next child will not be affected is, say, r, which is almost equal to unity.

The probability $x_s$ does not depend on which one of the s children is affected and is, in particular, the probability that there is an old germ cell mutation, given that one of the first $s - 1$ children is affected and the $s^{th}$ one is not.

Then, by Bayes' Theorem

$$x_s = \frac{qx_{s-1}}{qx_{s-1} + r(1-x_{s-1})}$$

From previous data and from the present study $x_1$ is small and r is very nearly equal to one; thus

$$x_s \simeq qx_{s-1}$$
$$x_s \simeq q^{s-1}.x_1$$

The probability, $y_s$, that the $(s+1)$th child will be affected is $px_s$ and, obviously, $y_s \simeq q^{s-1}y_1$. If there are $n_s$ families with s children of whom one is affected and who go on to have a further child, and if $a_s$ of these children are affected we may estimate $y_s$ by $a_s/n_s$ and $y_1$ by $a_s/(n_s q^{s-1})$, i.e. we can obtain an estimate of $y_1$ for each value of s. The variances of these estimates are $\{var(a_s)\}/(n_s^2 q^{2s-2})$ and, taking $a_s$ as a Poisson variable, this is approximately $a_s/n_s^2q^{2s-2}$. The expected value of this expression is $y_1/n_sq^{s-1}$, i.e. the variances for families of different sizes are approximately inversely proportional to $n_sq^{s-1}$ and thus the combined estimate for $y_1$ using data from all the families is

$$y_1 = \sum a_s / \sum n_s q^{s-1}$$

$y_1$ is the estimated risk for subsequent siblings in families with one affected child and no unaffected ones. For a family of size s with one affected child and $s - 1$ unaffected siblings the risk $y_s = q^{s-1}y_1$ can be estimated, given $y_1$ and q.

For families of size 1, 2, 3, . . . s, and just one affected case, the proportions with unrecognised germ cell mutations are $x_1 = y_1/p$, $qx_1$, $q^2x_1$ . . . $q^{s-1}x_1$.

### A4 Estimation of proportion of hereditary cases among sporadic unilateral retinoblastoma cases

There is at present no direct and generally applicable method of classifying sporadic unilateral retinoblastoma cases as hereditary and non-hereditary though advances in molecular genetics may soon make this possible. If we wish to estimate the proportion of gene-carriers in this group it is necessary to use information on their offspring. The method of estimation described below takes into account the fact that, for instance, a survivor with four unaffected children is less likely to be a gene carrier than one for whom there is only one unaffected child.

We denote the unknown proportion of unilateral sporadic cases that actually have the hereditary form by $\lambda$. This parameter and its standard error may be estimated using the method of maximum likelihood.

Let T be the total number of families in which one parent has unilateral sporadic retinoblastoma, n be the total number of children in a family, and r the number with retinoblastoma. Let u be the probability of retinoblastoma developing in a child if a parent is a gene carrier and v the probability otherwise. We assume that all children are followed up at least to the age by which all retinoblastomas will be diagnosed.

The likelihood of the observed data is

$$\Pi \binom{n}{r} [\lambda\, u^r (1-u)^{n-r} + (1-\lambda) v^r (1-v)^{n-r}],$$

the product being taken over the values of n and r for each of the T families.

The log likelihood L is

$$L = \sum[\log \binom{n}{r} + \log (f + g\lambda)]$$

$$\text{where } f = v^r (1 - v)^{n-r}$$

$$\text{and } g = u^r(1 - u)^{n-r} - v^r(1 - v)^{n-r}$$

The maximum likelihood estimate of $\lambda$, i.e. the estimated proportion of unilateral sporadic cases that are hereditary, is obtained by setting $\frac{dL}{d\lambda} = 0$ and solving this equation for $\lambda$:

$$\frac{dL}{d\lambda} = \sum (h + \lambda)^{-1}, \quad \text{where } h = f/g$$

and the summation is taken over the values of n and r for the T families.

For the present study the values of the n are shown in Table VII; all of the values of r, except one, are zero; in one family $r = n = 1$. Assuming the risk to the offspring is the same for unilaterally affected parents as it is for bilaterally affected parents, $u = 0.45$. The risk in the general population, v, is less than 1 in 20,000 and $1-v \simeq 1$.

For families where $r = 0$, $h \simeq 1/(0.55^n - 1)$
For the family with $r = n = 1$, $h \simeq 0$.

Table VII gives estimates of the numbers of families with 1, 2, 3, 4 and 5 children. Excluding the one affected child these numbers are 36, 41, 12, 3 and 1 respectively.

Writing $k_n = 1/(0.55^n-1)$, the estimated value of $\lambda$, the proportion of unilateral sporadic cases that are hereditary is the solution of $1/\lambda + 36/(k_1 + \lambda) + 41/(k_2 + \lambda) + 12/(k_3 + \lambda) + 3/(k_4 + \lambda) + 1/(k_5 + \lambda) = 0$.

Solving this equation gives an estimate of 0.0169 for the value of $\lambda$, with an estimated standard error, using the usual maximum likelihood method, of 0.0168. This standard error and that in the next paragraph are based on small numbers and it seems unlikely that the usual normal approximation is valid; they should be regarded as giving only a general idea of the precision of the estimates.

If we assume that the risk to the offspring of unilaterally affected parents with hereditary retinoblastoma is the same as that for the siblings of unilateral hereditary cases then, from the first part of Table IV, $u = 0.3$. Repeating the above calculations with this value of u gives an estimate of 0.0231 with a standard error of 0.0230.

### References

ABRAMSON, D.H., ELLSWORTH, R.M., KITCHIN F.D. & TUNG, G. (1984). Second non-ocular tumours in retinoblastoma survivors. *Ophthalmology*, **91**, 1351.

BONAITI-PELLIE, C., BRIARD-GUILLEMOT, M.L., FEINGOLD, J. & FREZAL, J. (1976). Mutation theory of carcinogenesis in retinoblastoma. *J. Natl Cancer Inst.*, **57**, 269.

BRIARD-GUILLEMOT, M.L., BONAITI-PELLIE, C., FEINGOLD, J. & FREZAL, J. (1974). Etude genetique du retinoblastome. *Humangenetik.*, **24**, 271.

CAVALLI-SFORZA, L.L. & BODMER, W.F. (1971). *The Genetics of Human Populations*. W.H. Freeman: San Francisco.

COWELL, J.K. (1991). The genetics of retinoblastoma. *Br. J. Cancer*, **63**, 333.

DER KINDEREN, D.J. (1987). *A new concept of oncogenesis with an evaluation in retinoblastoma.* University of Utrecht.

DER KINDEREN, D.J., KOTEN, J.W., NAGELKERKE, N.J.D., TAN, K.E.W.P., BEEMER, F.A. & DEN OTTER, W. (1988). Non-ocular cancer in patients with hereditary retinoblastoma and their relatives. *Int. J. Cancer*, **41**, 499.

DRAPER, G.J., SANDERS, B.M. & KINGSTON, J.E. (1986). Second primary neoplasms in patients with retinoblastoma. *Br. J. Cancer*, **53**, 661.

DRAPER, G.J., HEAF, M.M. & KINNIER WILSON, L.M. (1977). Occurrence of childhood cancers among siblings, and estimation of familial risks. *J. Med. Genet.*, **14**, 81.

DRYJA, T.P., RAPAPORT, J.M., JOYCE, J.M. & PETERSEN, R.A. (1986). Molecular detection of deletions involving band q14 of chromosome 13 in retinoblastomas. *Proc. Natl Acad. Sci. USA*, **83**, 7391.

FRIEND, S.H., BERNARDS, R., ROGELJ, S. & 4 others (1986). A human DNA segment with properties of the gene that predisposes to retinoblastoma and osteosarcoma. *Nature*, **323**, 643.

HARPER, P. (1988). *Practical Genetic Counselling* 3rd Edition. Wright: London.

HAWKINS, M.M., DRAPER, G.J. & SMITH, R.A. (1989). Cancer among 1,348 offspring of survivors of childhood cancer. *Int. J. Cancer*, **43**, 975.

KNUDSON, A.G. (1978). Retinoblastoma: a prototypic hereditary neoplasm. *Semin. Oncol.*, **5**, 57.

LEELAWONGS, N. & REGAN, D.J. (1968). Retinoblastoma: a review of ten years. *Am. J. Ophth.*, **66**, 1050.

MATSUNAGA, E. (1978). Hereditary retinoblastoma: delayed mutation or host resistance? *Am. J. Hum. Genet.*, **30**, 406–424.

MATSUNAGA, E. & OGYU, H. (1976). Retinoblastoma in Japan: follow-up survey of sporadic cases. *Jap. J. Ophth.*, **20**, 266.

MURPHREE, A.L. & BENEDICT, W.F. (1984). Retinoblastoma: clues to human oncogenesis. *Science*, **223**, 1028.

ONADIM, Z.O., MITCHELL, C.D., RUTLAND, P.C. & 5 others (1990). Application of intragenic DNA probes in prenatal screening for retinoblastoma gene carriers in the United Kingdom. *Arch. Dis. Ch.*, **65**, 651.

PETO, R., PIKE, M.C., ARMITAGE, P. & 7 others (1976, 1977). Design and analysis of randomized clinical trials requiring prolonged observation of each patient. I: Introduction and design. II: Analysis and examples. *Br. J. Cancer*, **34**, 585; **35**, 1.

SANDERS, B.M., DRAPER, G.J. & KINGSTON, J.E. (1988). Retinoblastoma in Great Britain 1969–80: incidence, treatment and survival. *Br. J. Ophth.*, **72**, 576–583.

SANDERS, B.M., JAY, M., DRAPER, G.J. & ROBERTS, E.M. (1989). Non-ocular cancer in relatives of retinoblastoma patients. *Br. J. Cancer*, **60**, 358.

SCHAPPERT-KIMMIJSER, J., HEMMES, G.D. & NIJLAND, R. (1966). The heredity of retinoblastoma. *Ophthalmologica*, **151**, 197.

TAMBOLI, A., PODGOR, M.F. & HORM, J.W. (1990). The incidence of retinoblastoma in the United States: 1974 through 1985. *Arch. Ophth.*, **108**, 128.

VOGEL, F. (1979). Genetics of retinoblastoma. *Hum. Genet.*, **52**, 1.

WIGGS, J., NORDENSKJOLD, M., YANDELL, D. & 11 others (1988). Prediction of the risk of hereditary retinoblastoma, using DNA polymorphisms within the retinoblastoma gene. *N. Engl. J. Med.*, **318**, 151.

YANDELL, D.W., CAMPBELL, T.A., DAYTON, S.H. & 6 others (1989). Oncogenic point mutations in the human retinoblastoma gene: their application to genetic counselling. *N. Engl. J. Med.*, **321**, 1689.